# Widespread and Biologically Driven Sex Disparities in Polygenic Risk Prediction Across Complex Traits

**DOI:** 10.21203/rs.3.rs-9153854/v1

**Published:** 2026-04-29

**Authors:** Akl Fahed, Xingyu Chen, Tingfeng Xu, Yang Sui, Kelvin Supriami, Min Seo Kim, Injeong Shim, Jennifer Halford, Kang Yu, Xinyu Zhou, Qiuli Chen, Jing Hao, Shaoqi Wang, Kewei Zhao, Shuangqiao Liao, Jichen Wen, Jiaqi Ding, Xinyu Zhu, Weixiang Zhao, Junyu Yang, Yu Zhang, Meier Chen, Komal Zaib, Bitao Zhong, Fei Wang, Whitney Hornsby, Kaavya Paruchuri, Pradeep Natarajan, Minxian Wang

**Affiliations:** Massachusetts General Hospital; Massachusetts General Hospital/ Broad Institute of MIT and Harvard/ Beijing Institute of Genomics (China National Center for Bioinformation); Beijing Institute of Genomics, Chinese Academy of Sciences. (China National Center for Bioinformation); Broad Institute of MIT and Harvard; Massachusetts General Hospital/ Broad Institute of MIT and Harvard; Broad Institute of MIT and Harvard; Massachusetts General Hospital; Massachusetts General Hospital, Broad Institute of MIT and Harvard; Beijing Institute of Genomics (China National Center for Bioinformation); Beijing Institute of Genomics (China National Center for Bioinformation); Beijing Institute of Genomics (China National Center for Bioinformation); Beijing Institute of Genomics (China National Center for Bioinformation); Beijing Institute of Genomics (China National Center for Bioinformation); Beijing Institute of Genomics (China National Center for Bioinformation); Beijing Institute of Genomics (China National Center for Bioinformation); China National Center for Bioinformation; Beijing Institute of Genomics (China National Center for Bioinformation); Beijing Institute of Genomics (China National Center for Bioinformation); Beijing Institute of Genomics (China National Center for Bioinformation); Beijing Institute of Genomics (China National Center for Bioinformation); Beijing Institute of Genomics (China National Center for Bioinformation); Beijing Institute of Genomics (China National Center for Bioinformation); Beijing Institute of Genomics (China National Center for Bioinformation); Beijing Institute of Genomics (China National Center for Bioinformation); Beijing Institute of Genomics (China National Center for Bioinformation); Massachusetts General Hospital; Broad Institute; Massachusetts General Hospital; Beijing Institute of Genomics (China National Center for Bioinformation)

## Abstract

Polygenic risk scores (PRS) are increasingly used for disease prediction, yet their performance equity across sexes remains unclear. We evaluated sex differences in PRS performance using 3,263 scores across 145 traits from the PGS Catalog in 409,440 UK Biobank participants. Sex-differential prediction was widespread and trait-specific, affecting 15 of 64 (23%) of diseases and 43 of 81 (53%) of quantitative traits. Female-favoring performance was enriched in autoimmune and endocrine traits, whereas cardiometabolic traits more often favored males. Discovery GWAS sex imbalance partially explained disease-level disparities (R^2^ = 0.36), whereas quantitative traits showed minimal association. Notably, sex differences in predictive performance strongly correlated with differences in SNP-based heritability from sex-stratified GWAS (R^2^ = 0.81 for diseases; R^2^ = 0.58 for quantitative traits). In contrast, PRS estimates were highly consistent across seven construction methods (intraclass correlation coefficient = 0.93), indicating limited methodological influence. These findings demonstrate that sex disparities in PRS performance are common and largely reflect underlying genetic architecture rather than analytic artifacts, highlighting the need for sex-aware GWAS design and PRS modeling to ensure equitable clinical implementation.

Polygenic risk scores (PRS), which aggregate the effects of thousands of genetic variants identified through genome-wide association studies (GWAS), have emerged as powerful tools for quantifying individual genetic predisposition to complex traits and diseases.^1^ By weighing risk alleles through effect size estimates from large-scale GWAS, PRS have demonstrated clinical potential by enabling disease prediction and risk stratification.^2–4^ However, the generalizability of PRS remains constrained across ancestral populations, with significantly reduced predictive accuracy in non-European ancestries, which are often underrepresented in genetic studies.^5,6^

Beyond ancestral disparities in representation, pronounced sex differences in disease prevalence persist across numerous traits. Coronary artery disease (CAD) exemplifies this divergence. Male sex is a risk factor for CAD, and females have delayed disease onset by 7–10 years compared to males^7^, yet females experience higher mortality rates post-myocardial infarction.^8^ Similarly, autoimmune diseases like rheumatoid arthritis show 3:1 female-to-male prevalence^9^, while neuropsychiatric disorders such as depression demonstrate sex-specific symptom profiles.^10^ These epidemiological disparities arise from multifaceted biological mechanisms, including genotype by sex (GxS) interactions^11^, X-chromosome dosage effects^12^, sex hormone-mediated gene regulation^13^, and sex-related environmental exposures^14^.

Despite these established sex differences, current PRS frameworks often overlook sex-specific genetic architectures. Most GWAS meta-analyses either inadequately stratify by sex or assume linear additive effects across sex,^15–17^ and PRS construction methods lack modelling of sex-specific effect sizes between sexes, potentially biasing predictions. For instance, prior analyses of CAD using a multiancestry polygenic risk score (GPS_Mult_) from our group^18^ revealed substantial sex-based prediction disparities. This imbalance likely stems from both biological factors, including underrepresentation of sex-specific loci in GWAS, and methodological limitations, including pervasive sample ascertainment bias^19^ and insufficient modeling of gene-by-sex interactions^20^.

As polygenic scores move towards clinical implementation, the absence of systematic evaluation of sex bias poses a critical barrier to equitable use^21,22^. A sex-blind approach may exacerbate disparities, yet no study has comprehensively quantified these differences or clarified their methodological and biological sources. Filling this gap is critical for allowing fair and effective clinical translation of PRS.

To address these gaps, we used more than three thousand PRS from the PGS Catalog in conjunction with UK Biobank data to broadly assess the prevalence and magnitude of sex-dependent predictive disparities. We then leveraged GWAS data from the UK Biobank training dataset and employed seven PRS methods to investigate how GWAS sample size differences, methodological biases, and biological heterogeneity at sex-specific loci drive these disparities.

## Method

### Study Population and Outcome Definitions

We utilized data from the UK Biobank^23^ under application IDs 7089 and 89885, which granted access to anonymized genetic and clinical data. Standard UKB quality control procedures were applied. Specifically, individuals exhibiting discrepancies between self-reported sex (UK Biobank Field 31) and genetically inferred sex (Field 22001) were excluded. Additionally, participants were removed if they presented an individual-level genotype missingness rate exceeding 5% (Field 22005), were outliers in heterozygosity or missing rate (Field 22027), or exhibited sex chromosome aneuploidy (Field 22019). After applying these QC steps and restricting analyses to genetically unrelated individuals of European ancestry (kinship coefficient < 0.0884)^24^, a total of 409,440 participants remained for further analysis. The cohort was split into independent training (50%), tuning (25%), and testing (25%) sets to enable unbiased model development, parameter optimization, and final evaluation of PRS performance.

Disease outcomes were defined using a combination of self-report and linked health records (Supplementary Table 1), and quantitative traits were centrally curated by the UKB.

### Curation of Polygenic Risk Scores for Evaluation

To systematically investigate whether PRS exhibit widespread sex differences in their predictive performance, we used the PGS Catalog to assess sex bias in PRS predictive performance.^25^ A total of 5,008 PRS (as of October 2024) were retained for subsequent analysis. We harmonized SNP identifiers between PGS Catalog weights and UK Biobank genotypes using standard coordinate and allele matching; scores with <80% variant overlap were excluded. For PRS with variant matching rates more than 0.8, we utilized all matched variants to calculate the PRS scores. This standardized procedure was uniformly applied across all PGS Catalog entries, ensuring consistency in our analysis involving over 5,000 publicly available PRSs.

From the 692 phenotypes in the PGS Catalog, we derived a curated set by applying exclusion criteria to ensure alignment with UK Biobank phenotypes and minimize conceptual or ethical ambiguities. We excluded traits represented by three or fewer PRS, those lacking direct UK Biobank counterparts, and descriptors that were broad or imprecise. Additional exclusions included disease subtypes nested within broader diagnoses, family-history and virology or serology traits, imaging-derived or procedural measures, isolated symptoms or behavioral items, narrowly defined dietary exposures, sex-specific conditions such as prostate or ovarian cancer, lifestyle and socioeconomic factors, and traits potentially raising ethical concerns in the context of sex differences. This yielded 145 phenotypes (64 diseases and 81 quantitative traits) for downstream PGS Catalog analyses (Supplementary Table 1).

For each retained weight file, polygenic scores were generated in PLINK2(v2.0.0-a.6.5LM)^26^ by multiplying the dosage of every risk allele by its assigned effect size and aggregating these weighted values across all variants for each individual in the UK Biobank.

### Evaluation of Sex Differences in Published Polygenic Risk Scores

After obtaining a comprehensive list of published PRS from the PGS Catalog, we evaluated their sex-specific predictive performance at both the score and trait levels. For each individual score, sex-stratified regression models were fitted in the complete UK Biobank data, and differences between males and females were assessed using a Z test. Scores were classified as female-biased, male-biased, or unbiased based on the direction and significance of effect differences. At the trait level, all scores for a phenotype were jointly modeled in the tuning set to derive a weighted ensemble score for phenotype prediction (ensemble trait-level PRS), which was then applied to the testing set for sex-specific evaluation.

### Association of Discovery Sample Size with Sex-Differential PRS Performance

To assess whether discovery cohort composition contributed to sex differences in PRS performance, GWAS sample sizes for each PGS Catalog score were obtained from the REST application programming interface (API)^25^, which supports batch access to curated score metadata, and were complemented by manual curation from primary publications when API fields were unavailable. Sex-specific counts were derived from reported male proportions or case-control sample size, expressed as effective sample sizes (as twice the harmonic mean of the case and control populations) for binary traits and total sample sizes for quantitative traits. Female-to-male ratios of discovery sample size were then compared with corresponding PRS performance ratios using regression analyses. If there are multiple scores for a trait, the sample size and the PRS performance were averaged. Outliers (Cook’s distance > 4/n or studentized residual > 3) were excluded from model fitting but retained in plots.

### Sex Stratified and Sex Agnostic GWAS Analysis

To generate GWAS summary statistics for downstream dissection of sex differences in polygenic risk scores, we conducted both sex-agnostic and sex-stratified genome-wide association studies (GWAS) using imputed genotype data from the UK Biobank. Analyses were performed with REGENIE (v3.2.9)^27^ following its standard two-step procedure.

Step 1 fitted whole-genome regression models under a leave-one-chromosome-out (LOCO) scheme using high-quality genotyped variants: minor allele frequency (MAF) > 1%, minor allele count (MAC) > 100, genotyping call rate > 99%, Hardy–Weinberg equilibrium P > 1×10^−15^, missingness < 10%, and linkage disequilibrium (LD) pruning using 1,000-variant windows, 100-variant sliding windows, and an r^2^ threshold < 0.8.

In the second step, single-variant association tests were performed using linear regression with LOCO-based predictions included as offsets. Sex-agnostic GWAS adjusted for age, age^2^, sex, assessment center, genotyping array, and 10 ancestry PCs; sex-stratified GWAS applied the same covariates, excluding sex.

### Assessment of Methodological Differences in PRS Construction

To evaluate whether the observed sex differences in PRS were influenced by the algorithms used to derive PRS, we analyzed 58 sex-biased traits using a sex-agnostic GWAS in the UK Biobank training set, using the same training, tuning, and testing partitions as in the primary analyses.

PRS were then constructed using seven widely adopted algorithms: clumping and thresholding (P+T), LDpred2, Lassosum2, SBayesR, SBLUP, SDPR, and PRS-CS^26,28–36^. Method parameters are provided in Supplementary Table 6. Linkage disequilibrium (LD) reference panels were derived from 503 unrelated European individuals from the 1000 Genomes Project Phase 3 dataset^37^ (MAF > 1%), ensuring consistency across all methods. Only HapMap3 variants were included in PRS construction^38^.

For each trait and method, the parameters of all candidate PRS were tuned in the tuning subset and evaluated in the testing dataset. Consistency of sex-bias estimates across methods was assessed by intraclass correlation (*ICC*s) with 1,000-bootstrap 95% CIs using the pingouin package (v0.5.4)^39^ in Python (v3.10).

### Heritability Estimation

SNP-based heritability (h^2^) was estimated using linkage disequilibrium score regression (LDSC)^40^ applied to GWAS summary statistics, and calculated as

$$h_{g}^{2}=\frac{\sigma_{g}^{2}}{\sigma_{g}^{2}+\sigma_{\varepsilon }^{2}}$$

where, $\sigma_{g}^{2}$ and $\sigma_{\varepsilon }^{2}$ are the estimates of the additive genetic and residual variance. Analyses were restricted to well-imputed HapMap3 variants. Chi-square statistics were regressed on LD scores computed from the 1000 Genomes European reference panel. Heritability estimates for binary traits were transformed from the observed scale to the liability scale, using the sex-specific population prevalence of the trait, under the assumption of an underlying normal distribution of liability to the considered trait, as described previously.^41^

### Statistical Analysis

All analyses were conducted under a prespecified, uniform analytic framework to ensure consistency across PGS Catalog, ensemble, and de novo PRS analyses. For binary outcomes, logistic regression models were fitted with standardized PRSs as predictors. For quantitative traits, linear regression was applied. Covariates included age, sex, assessment center, genotyping array, and the first 10 principal components of ancestry, with sex excluded in sex-stratified analyses. To minimize confounding from population structure, all PRSs were residualized by the first 10 genetic principal components before association testing.

Sex differences in predictive performance were quantified using a two-sided Z test comparing sex-specific regression coefficients and their standard errors:

$$\frac{\beta_{\text{female}}-\beta_{\text{male}}}{\sqrt{{SE}_{\text{female}}^{2}+{SE}_{\text{male}}^{2}}}$$

This test evaluates the null hypothesis that the predictive effects of PRS are equal between sexes (H_0_: *β*_female_ = *β*_male_) against the alternative hypothesis that they differ (H_1_: *β*_female_ ≠ *β*_male_). This procedure was applied consistently across all analytic stages, including PGS Catalog scores, ensemble trait-level PRSs, and UK Biobank–derived PRSs constructed from multiple algorithms.

For secondary comparisons of genetic parameters between male- and female-biased traits, we used the Mann–Whitney U test to evaluate whether the median difference between groups differed significantly from zero (*H*_*0*_: Δ = 0; *H*_*1*_: Δ ≠ 0).

Sex bias in predictive performance was represented as the difference in these metrics between females and males. Multiple testing correction was performed using the Benjamini–Hochberg false discovery rate (FDR) procedure, with *FDR*-adjusted *p* < 0.05 considered statistically significant. All statistical tests were two-sided. Regression modeling and data visualization were performed in R version 4.1.2 (R Foundation for Statistical Computing) with the bigsnpr^32^, ggplot2^42^, and data.table packages. Analyses reporting include point estimates and 95% confidence intervals for all primary effect size comparisons.

## Results

### Widespread Sex Bias in Polygenic Risk Score Performance

**Figure 1:**
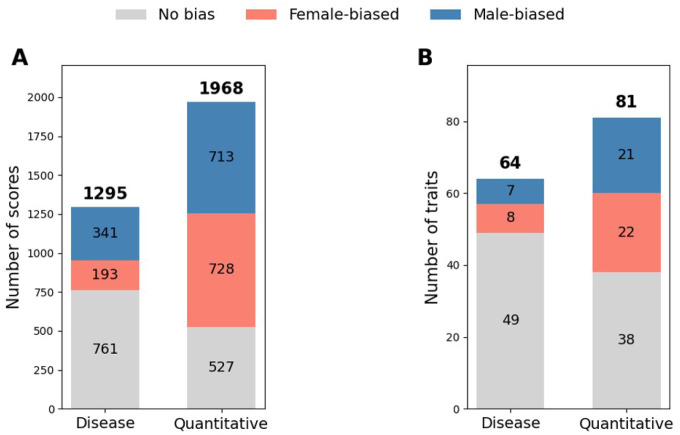
Distribution of Sex Bias in Polygenic Risk Score Performance by Score and Trait Stacked bars summarize the number of PGS Catalog scores evaluated in the UK Biobank cohort (N=409,440 after quality control; European ancestry). Panel A: score-level results for disease and quantitative scores (No bias=gray; Female-biased=red; Male-biased=blue). Panel B: trait-level aggregation. Numbers above bars denote totals; in-bar labels denote category counts. Sex differences in scores were assessed using a 2-sided Z test comparing sex-stratified regression coefficients with *FDR*<0.05, and traits were determined by ensembling all scores and evaluating them with the same Z test strategy. PRS values were standardized to a mean of 0 and a standard deviation of 1; regression covariates included age, sex, genotyping array and top 10 genetic principal component.

Using PRS from the PGS Catalog applied to the UK Biobank, we observed widespread and trait-specific sex differences in polygenic risk prediction across both diseases and quantitative traits. Figure 1 summarizes the distribution of sex bias across scores (left) and traits (right). Among 1295 disease scores, 41.2% showed significant sex bias (193 female-biased; 341 male-biased). Among 1968 scores for quantitative traits, 73.2% were biased (728 female-biased and 713 male-biased). Aggregated at the trait level, 15 (23.4%) of 64 disease traits and 43 (53.1%) of 81 quantitative traits displayed significant sex differences in performance, respectively.

**Figure 2:**
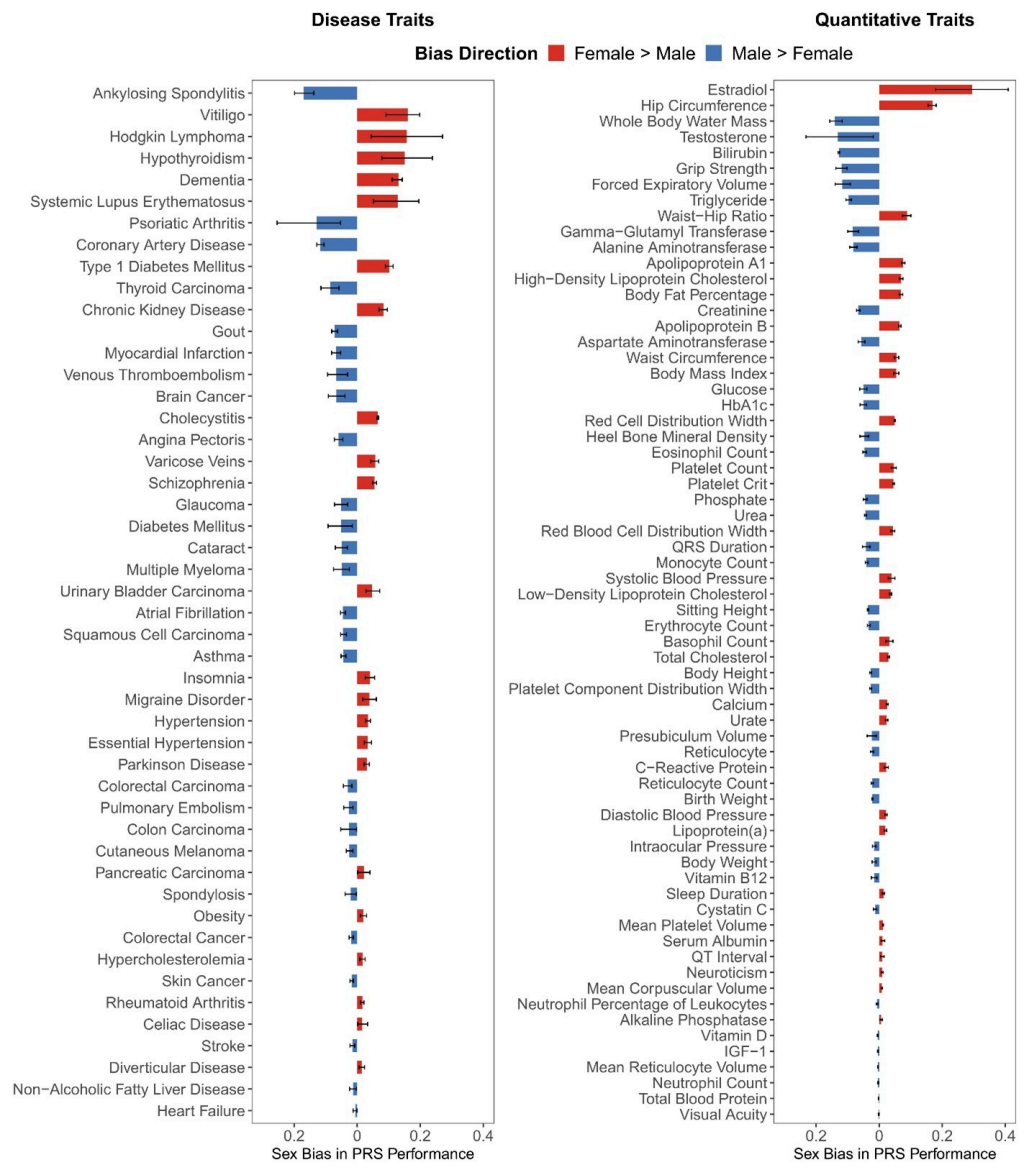
Trait-Level Sex Bias in PRS Performance in the PGS Catalog For each trait, multiple PRSs were combined into a single ensemble score using multivariable regression in the UK Biobank tuning set. This ensemble score was then evaluated separately in males and females; the difference in regression coefficients *β*_female_ − *β*_male_ was used to represent sex bias. Positive values indicate stronger predictive performance in females, while negative values indicate stronger predictive performance in males. Error bars represent 95% confidence intervals for the sex-difference estimates. Traits are ordered by absolute effect size to facilitate comparison. PRS values were standardized to a mean of 0 and a standard deviation of 1; regression covariates included age, sex, genotyping array and top 10 genetic principal component.

To further characterize the magnitude and direction of these effects, we extended the trait-level summary to display effect size estimates and 95% confidence intervals for each trait (Figure 2). This analysis revealed marked heterogeneity across traits, with female-favoring prediction observed for autoimmune and endocrine diseases such as hypothyroidism and type 1 diabetes, whereas male-favoring prediction was evident for cardiovascular diseases, including coronary artery disease and atrial fibrillation. Other traits, such as inguinal hernia, are also male-biased. Among quantitative traits, hormone- and body composition–related measures such as estradiol, testosterone, and body fat showed the strongest sex divergence, typically favoring the biologically corresponding sex, while most hematologic and metabolic biomarkers exhibited smaller but consistent male advantage.

Overall, these findings demonstrated that sex differences in PRS performance are both pervasive and trait-specific, affecting a wide spectrum of diseases and quantitative phenotypes. Full per-score and per-trait statistics, including confidence intervals and significance levels, are provided in Supplementary Table 3 and Supplementary Table 4.

### Sample Size Composition of GWAS Contributes to Sex Bias

**Figure 3:**
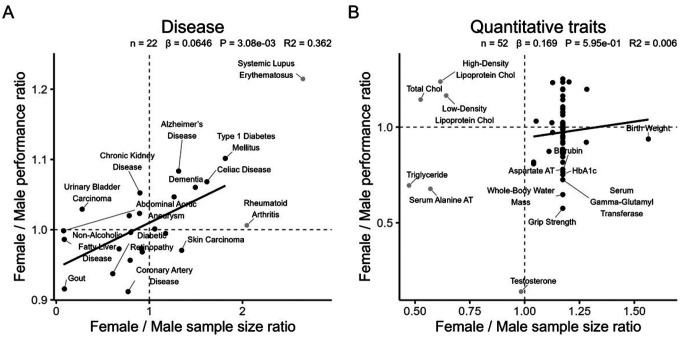
Association Between Discovery Sample Size Imbalance and Sex Differences in PRS Performance Scatterplots show the relationship between female-to-male ratios of GWAS discovery sample size (X-axis) and corresponding female-to-male ratios of PRS predictive performance (Y-axis) for disease (left) and quantitative (right) traits. Each point represents one trait derived from the PGS Catalog. PRS values were standardized to a mean of 0 and a standard deviation of 1

To assess whether imbalanced sex representation in the discovery GWAS used to derive PRS contributes to sex-related differences in PRS predictive performance, we examined the relationship between female-to-male ratios of GWAS sample sizes and corresponding ratios of PRS predictive performance for each trait (Figure 3). For disease traits, a positive correlation was observed (*β* = 0.0646, *P*-value = 3.08 × 10^−3^, *R*^2^ = 0.36), indicating that PRS derived from a larger female GWAS sample size tended to perform better in females. In contrast, quantitative traits showed no meaningful association (*β* = 0.169, *P*-value = 0.60, *R*^2^ = 0.006). These findings suggest that sample-size imbalance in GWAS discovery cohorts partially drives the PRS prediction bias between sexes, consistent with the expectation that larger sample sizes yield more accurate estimates of allele effects, thereby improving prediction power.

### Consistency of Sex Bias Across PRS Methods

Building on the PGS Catalog-wide characterization of sex differences, and complementary to the analysis of discovery sample composition, we evaluated whether the observed biases depend on the choice of PRS algorithm. We focused on 58 traits that were significantly sex-biased in the PGS Catalog analysis and generated polygenic scores using 7 widely used methods.

**Figure 4:**
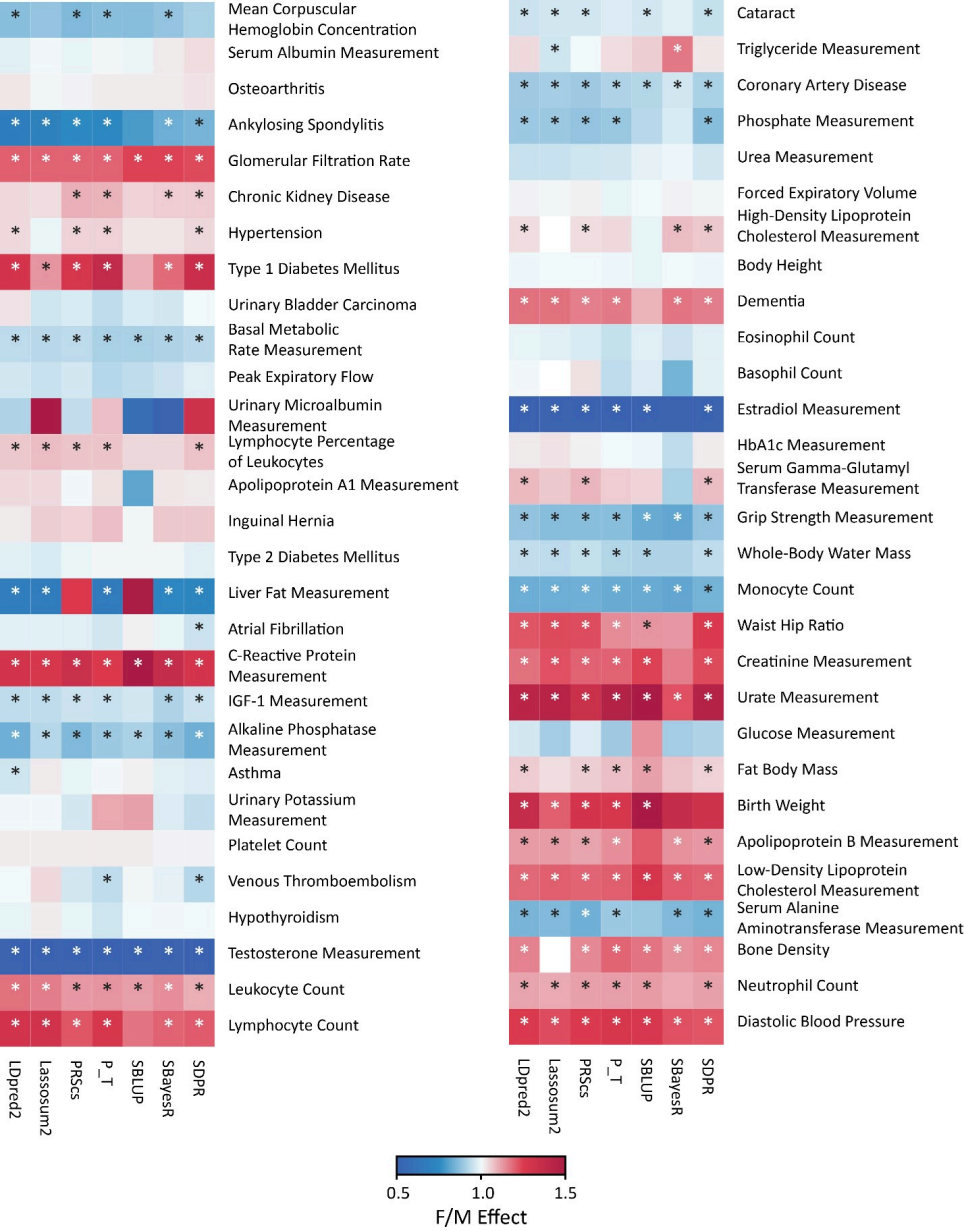
Heatmap of Sex Bias in PRS Performance Across 58 Traits and 7 Methods Each cell displays the female-to-male performance ratio for a given trait–method pair (*β*_female_/*β*_male_), among 58 traits and 7 methods. Warmer vs cooler colors indicate ratios >1 vs <1. Asterisks denote pairs with significant sex differences by Z test comparing sex-stratified regression coefficients with *FDR* < 0.05. PRS values were standardized to a mean of 0 and a standard deviation of 1; regression covariates included age, sex, genotyping array and top 10 genetic principal component.

Across methods, trait-level estimates were relatively concordant (Figure 4). Patterns in the trait-by-method matrix clustered by trait rather than by method, and no algorithm consistently amplified or attenuated female- or male-favoring effects. We used the Intraclass Correlation Coefficient (ICC) to assess consistency across methods. Agreement across the 7 method-specific estimates was high, with an intraclass correlation of *ICC*(2,k)=0.93 (95% CI, 0.90–0.96; P<.001), and virtually identical results for *ICC*(1,k) and *ICC*(3,k) (Supplementary Table 8).

These findings indicate that sex-differential PRS performance is robust to algorithmic choice and thus more likely reflects trait-specific biology rather than method artifacts. Detailed performance metrics and statistical results are provided in Supplementary Table 7.

### Potential Role of Genetic Architecture in Shaping Sex Differences

To examine whether sex differences in PRS performance reflect underlying differences in genetic architecture, we conducted sex-stratified GWAS for traits that exhibited significant sex bias in PRS performance, including 15 disease traits and 43 quantitative traits. Using sex-specific GWAS summary statistics from the UK Biobank, heritability was estimated with linkage disequilibrium score regression (LDSC)

**Figure 5:**
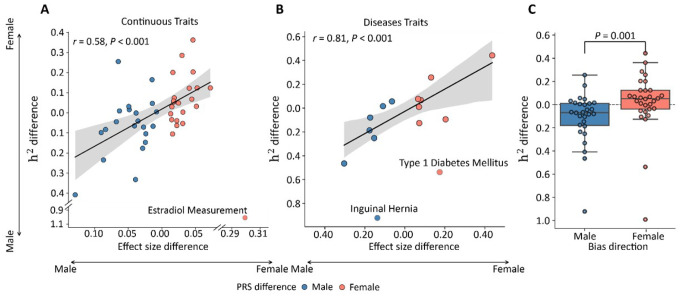
Association Between Sex Differences in Polygenic Risk Score Performance and Heritability A,B, Scatterplots show the association between sex differences in polygenic risk score (PRS) predictive effect (X-axis) and SNP-based heritability (Y-axis) estimated from sex-stratified GWAS in the UK Biobank. The X-axis represents the difference in PRS effect size between males and females (effect size difference = *β*_male_ – *β*_female_), and the Y-axis represents the difference in heritability (h^2^ difference = h^2^_male_ – h^2^_female_). Positive values indicate higher performance or heritability in males, negative values indicate higher performance in females. Panels A and B display results for quantitative and disease traits, respectively. **C**, Boxplot of the difference in heritability stratified by PRS prediction bias. The X-axis indicate bias category, Y-axis indicate SNP-based heritability difference. The significance between groups was tested using a Mann–Whitney U test (P = 1.312 × 10^−3^).

We observed that the magnitude of sex differences in PRS performance was significantly correlated with the gap in SNP-based heritability between sexes (Figure 5). Among disease and quantitative traits, the correlation was strong. The Pearson correlation was 0.81 (P < 0.001) and 0.58 (P < 0.001) for disease and quantitative traits, respectively. Notably, several traits, such as type 1 diabetes, deviated from this overall trend, suggesting that additional mechanisms beyond differences in total heritability may contribute to sex-specific prediction disparities.

To further assess whether overall heritability patterns differ systematically between male- and female-biased traits, we compared the distributions of heritability difference across traits classified by PRS bias direction. Traits whose PRSs performed better in males exhibited significantly higher male-to-female heritability than those favoring females (P = 1.312 × 10^−3^; Figure 5C). This result supports the notion that, on average, higher SNP-based heritability corresponds to stronger PRS predictive performance, consistent with sex-dependent differences in polygenic architecture.

Collectively, these findings indicate that sex-dependent genetic architecture is a strong driver for sex differences in PRS performance.

## Discussion

This study demonstrates that sex differences in PRS performance are both widespread and biologically grounded. Prior PRS implementation studies have noted isolated instances of sex differences in risk stratification^7,18,43^, yet none have systematically quantified their prevalence across thousands of polygenic scores and diverse trait categories. Our analysis provides population-level evidence that sex disparities are both common and trait-specific, emphasizing the need for sex-aware evaluation before clinical deployment.

Leveraging more than 3,000 publicly available PRS and newly generated scores using published GWAS and multiple methods, we found that disparities between males and females were common across diverse traits and partly attributable to an imbalance in discovery GWAS sample composition, while algorithmic choice had minimal influence. Autoimmune and endocrine conditions tended to show stronger prediction in females, whereas cardiometabolic conditions favored males, mirroring well-established patterns of sexual dimorphism. This is aligned with the previous study.^7,18,43^ Notably, the association between GWAS sample size composition and sex differences in PRS performance was primarily observed for disease traits but not for quantitative traits. One possible explanation is that, for disease phenotypes, GWAS sample size may partly reflect underlying disease characteristics such as prevalence and heritability, which influence statistical power for variant discovery and downstream PRS construction.^44^ In contrast, the sample size for quantitative traits is typically determined by the availability of phenotype measurements rather than the genetic architecture of the trait itself, which may explain the lack of a comparable relationship. These findings indicate that sex differences in PRS performance are unlikely to arise from analytic artifacts alone and underscore the importance of sex-balanced GWAS designs and sex-aware PRS frameworks.

Biological factors emerged as a stronger and more consistent driver. Sex-stratified GWAS demonstrated that differences in PRS performance closely mirrored differences in SNP-based heritability, with stronger correlations for disease traits. Traits with higher heritability in one sex consistently showed stronger PRS prediction in that sex, pointing to systematic differences in polygenic architecture. These observations align with prior evidence of sex-dependent heritability, variant-effect heterogeneity, and hormone-related gene regulation, suggesting that sex differences in PRS reflect genuine biological divergence in genetic architecture rather than analytic artifacts.^11,20,45^ This convergence across PGS Catalog scores, UK Biobank PRS scores, and sex-stratified GWAS reinforces the biological foundation of sex-biased prediction.

These findings have important implications for clinical translation. As PRS are increasingly incorporated into preventive clinical genomics programs, unrecognized sex differences may lead to systematic misclassification of genetic risk. Female risk for cardiometabolic diseases may be underestimated, while male risk for autoimmune or endocrine disorders may be misestimated, with potential consequences for screening eligibility, preventive therapy allocation, and long-term monitoring. Routine evaluation of sex-stratified predictive effectiveness should therefore become a standard component of PRS validation prior to clinical adoption.

Our analysis was limited to autosomal variants in European ancestry participants, and X-chromosome and multi-ancestry analyses are warranted in further studies. Our analysis was also limited by incomplete metadata in the PGS Catalog API, as many studies did not report discovery sample sizes or sex composition, and large-scale manual curation was not feasible. Some underlying GWAS datasets were not publicly available, which may have reduced the completeness of our sample-size analysis.

Overall, this study provides the most comprehensive evaluation to date of sex-differential PRS performance across complex traits and demonstrates that these differences are pervasive and largely biologically driven. Addressing sex-dependent genetic architecture will be essential for fair and clinically meaningful implementation of polygenic risk scores.

## Supplementary Files

This is a list of supplementary files associated with this preprint. Click to download.

- SupplementaryTables.pdf
- SupplementaryFigure1.docx

## Data Availability

Individual-level data from the UK Biobank are available to qualified researchers through a formal application (https://www.ukbiobank.ac.uk). Summary-level GWAS statistics and PGS weights used in this study are publicly accessible from the PGS Catalog (https://www.pgscatalog.org). All derived PRS performance metrics and summary statistics are available upon reasonable request to the corresponding author.

## Code Availability

Code used for polygenic risk score construction, evaluation, and statistical analyses in this study is publicly available at https://github.com/xinyu-c9/SexdiffPRS.
